# Sex differences in lifespan extension with acarbose and 17‐α estradiol: gonadal hormones underlie male‐specific improvements in glucose tolerance and mTORC2 signaling

**DOI:** 10.1111/acel.12656

**Published:** 2017-08-22

**Authors:** Michael Garratt, Brian Bower, Gonzalo G. Garcia, Richard A. Miller

**Affiliations:** ^1^ Department of Pathology University of Michigan Medical School Ann Arbor MI 48109 USA; ^2^ University of Michigan Geriatrics Center Ann Arbor MI 48109 USA

**Keywords:** estrogen, life‐extension, testosterone

## Abstract

Interventions that extend lifespan in mice can show substantial sexual dimorphism. Here, we show that male‐specific lifespan extension with two pharmacological treatments, acarbose (ACA) and 17‐α estradiol (17aE2), is associated, in males only, with increased insulin sensitivity and improved glucose tolerance. Females, which show either smaller (ACA) or no lifespan extension (17aE2), do not derive these metabolic benefits from drug treatment. We find that these male‐specific metabolic improvements are associated with enhanced hepatic mTORC2 signaling, increased Akt activity, and phosphorylation of FOXO1a – changes that might promote metabolic health and survival in males. By manipulating sex hormone levels through gonadectomy, we show that sex‐specific changes in these metabolic pathways are modulated, in opposite directions, by both male and female gonadal hormones: Castrated males show fewer metabolic responses to drug treatment than intact males, and only those that are also observed in intact females, while ovariectomized females show some responses similar to those seen in intact males. Our results demonstrate that sex‐specific metabolic benefits occur concordantly with sexual dimorphism in lifespan extension. These sex‐specific effects can be influenced by the presence of both male and female gonadal hormones, suggesting that gonadally derived hormones from both sexes may contribute to sexual dimorphism in responses to interventions that extend mouse lifespan.

## Introduction

There is increasing recognition that lifespan‐extending manipulations can have sexually dimorphic effects on survival. Genetic impairments in several components of the insulin‐like growth factor‐1 (IGF1) signaling pathway have been shown to extend lifespan to a greater extent in female mice than in males (Garratt *et al*., 2017), with reduced IGF1 signaling sometimes generating significant lifespan extension only in females (Holzenberger *et al*., 2003; Bokov *et al*., 2011; Svensson *et al*., 2011; Xu *et al*., 2014). Reduced mTORC1 signaling has also been reported to extend lifespan to a greater degree in females (Lamming *et al*., 2012; Miller *et al*., 2014; Zhang *et al*., 2014; Garratt *et al*., 2016). By contrast, several different pharmacological treatments, including aspirin, nordihydroguaiaretic acid, acarbose (ACA), Protandim, and 17‐α estradiol (17aE2), extend mouse lifespan to a greater degree in males (Harrison *et al*., 2014; Strong *et al*., 2016). The causes for this sexual dimorphism in lifespan extension are largely unknown (Austad & Bartke, 2015).

ACA and 17aE2 each have reproducible and robust effects on male median and maximum lifespan, with noticeably smaller or undetectable effects in females. ACA is a glucosidase inhibitor that slows down carbohydrate digestion and reduces postprandial glucose spikes (Harrison *et al*., 2014). Treatment with ACA can extend male lifespan by around 20%, but leads to much smaller, although still significant, 5% extension in females. As this drug controls excursions in blood glucose levels, and is used to treat type II diabetes, this sexually dimorphic lifespan response might suggest that lifespan in male mice is more sensitive to alterations in blood glucose fluctuations than that of females. 17aE2 is a nonfeminizing steroid that has a reduced affinity for the classical estrogen receptors (Harrison *et al*., 2014). Treatment of mice with 17aE2 can extend male lifespan by 19% without any noticeable effects in females (Strong *et al*., 2016). This striking sex specificity of the lifespan effects of 17aE2 might suggest that some aspect of estrogenic signaling, outside of the effects of classical estrogen receptor (ER) signaling, which require strong binding affinity to ER, might be particularly beneficial for males but not females. It has further been suggested that 17aE2 might have particular actions in the brain, where it can bind to a nonclassical ER receptor ER‐X, which can modulate MAPK/ERK signaling (Toran‐Allerand *et al*., 2002, 2005). Treatment of 16‐month‐old male C57BL/6 mice with 17aE2 ameliorates metabolic and inflammatory dysfunction, suggesting that this steroid may have metabolic benefits (Stout *et al*., 2016), although this report did not include treated females as a comparison group.

A potential role of ACA and 17aE2 in improving metabolic dysfunction in males, specifically, is also consistent with sex‐specific patterns of glucose–insulin homeostasis and metabolism characteristic of mice and humans. Males and females differ in their production of hormones involved in the regulation of glucose metabolism, and can differ in insulin sensitivity and glucose homeostasis (Legato, 2010). In particular, male mice of a variety of different strains have been reported to have lower insulin sensitivity and lower rates of glucose clearance when compared to females (Macotela *et al*., 2009; Bonaventura *et al*., 2013; Sadagurski *et al*., 2014; Shivaswamy *et al*., 2014). Furthermore, genetic inhibition of several components of the insulin signaling cascade (IRS2, mTORC2), which impair glucose homeostasis, greatly increases mortality rates of males with noticeably smaller effects on female survival (Selman *et al*., 2008; Lamming *et al*., 2014b). Thus, treatments that improve glucose control might plausibly provide greater benefits to males. The underlying causes for sex differences in glucose homeostasis are not fully understood, but sex‐specific gonadal hormone production has been implicated. Testosterone, in some instances, can reduce insulin sensitivity, and 17‐β‐estradiol can provide benefits, with the latter expected to contribute to alterations in glucose homeostasis and elevated adiposity after menopause (Mauvais‐Jarvis, 2015). However, the effects of testosterone and 17‐β‐estradiol on insulin sensitivity and glucose metabolism can be context dependent, with each hormone reported to have opposing effects in some instances (Geer & Shen, 2009).

In this study, we tested whether male lifespan extension with ACA and 17aE2 is associated with benefits to males in terms of improved glucose homeostasis, and whether these effects differ from those in females. Because male and female lifespan and glucose homeostasis are differentially affected by changes in mTOR signaling (Lamming *et al*., 2012, 2014b), we were further interested in whether changes in mTOR signaling may be implicated in these responses. To provide additional insight into the hormonal underpinnings of this sex specificity, we also examined responses to ACA and 17aE2 in castrated males and ovariectomized (OVX) females. This endocrine manipulation allowed us to test whether sex‐specific responses to these drugs were related to the presence of male or female gonads and associated sex‐specific hormone production.

## Results

### Hormones regulated similarly in both sexes

**Figure 1:**
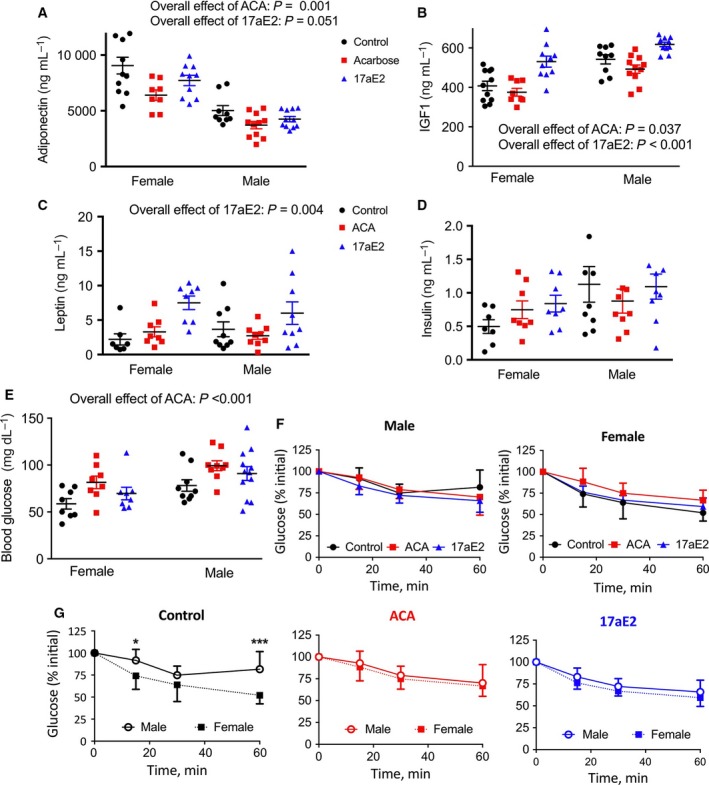
Alterations in metabolic hormones (A–D) and insulin sensitivity (E–G) in male and female mice treated with ACA or 17aE2. Hormone levels (A–D) were assessed in plasma samples collected from 12‐month‐old mice (*n *= 7–12 per sex, per group). Insulin tolerance tests were conducted in 9‐month‐old mice (*n *= 8 per group per sex). F&G show the same set of data, plotted to show effects of treatment on sex differences (G) or the overall impact of treatment within each sex (F). Statistics showing overall effects represent the *P*‐value for a treatment parameter in a two‐way ANOVA that also included a parameter for sex. * represents *P* < 0.05; *** presents *P* < 0.005 from a Student's *t*‐test.

Several hormones involved in glucose control, which have previously been reported to show sex differences in circulating concentration, were influenced by ACA and/or 17aE2, but responses were similar in both sexes. Plasma adiponectin was higher in females than in males and was reduced by ACA and 17aE2 to a similar degree in both sexes (Fig. 1A). Plasma IGF1 concentration was higher in males and was reduced by ACA and increased by 17aE2 (Fig. 1B). Plasma leptin levels were similar between the sexes and were increased by 17aE2 (Fig. 1C). There was no overall effect of ACA or 17aE2 on fasting insulin levels (Fig. 1D), nor was there a significant interaction between sex and drug treatment on fasting insulin levels for either ACA or 17aE2 compared to control (*P *> 0.1 in each case). Fasting plasma glucose levels are elevated by ACA in both sexes (Fig. 1E), presumably a consequence of the slowed breakdown of ingested starch, replicating findings from an earlier cohort (Harrison *et al*., 2014).

### Sex‐specific changes in insulin sensitivity with ACA and 17aE2

To test for sex‐specific responses in insulin sensitivity with ACA and 17aE2 more directly, we conducted insulin tolerance tests (Fig. 1F). Males and females showed significantly different changes in insulin sensitivity with ACA or 17aE2 treatment, as highlighted by the significant sex*treatment interaction terms for change in glucose after insulin injection (Table 1). This generally reflected a slight improvement in insulin sensitivity in males and a slight reduction in insulin sensitivity in females, although only the reduction in insulin sensitivity for females with ACA is significant (Table 1). Also notable was that treatment with ACA and 17aE2 effectively suppressed the sex differences in insulin sensitivity often observable with this test. On the control diet, males showed less glucose responsiveness to an injection of insulin than females, a response reported previously (Macotela *et al*., 2009; Bonaventura *et al*., 2013; Sadagurski *et al*., 2017; Shivaswamy *et al*., 2014). By contrast, when males and females are treated with ACA and 17aE2, this sex difference disappeared (Fig. 1F,G; Table 1).

**Table 1:** Summary of metabolic traits showing a sex‐specific response to ACA and/or 17aE2, and effects of gonadectomy on these responses. Bold values denote significance at P < 0.05

	Effect of ACA	Effect of 17aE2	Sex by treatment interactions		Surgery by treatment interactions ACA		Surgery by treatment interactions 17aE2	
			ACA	17aE2	Castration	Ovariectomy	Castration	Ovariectomy
Insulin sensitivity (change in glucose)	Decreased in females (*P* = 0.02)	Increased in males (*P* = 0.06)	***P *** **= 0.036**	***P *** **= 0.030**	Not tested	Not tested	Not tested	Not tested
Glucose tolerance (AUC)	Increased in males	Increased in males	***P *** **= 0.033**	***P *** **= 0.003**	*P *= 0.14	*P *= 0.84	*P *= **0.020**	*P *= 0.35
Liver pNDRG1	Increased in males	Increased in males	***P *** **= 0.005**	***P *** **= 0.034**	***P *** **= 0.016**	***P *** **= 0.044**	*P *= 0.056	***P *** **= 0.049**
Liver pSGK1	Increased	Increased in males	*P *= 0.34	***P *** **= 0.001**	*P *= 0.18	*P *= 0.17	***P *** **= 0.003**	*P *= 0.055
Liver pAKT473	Increased	Increased in males	*P *= 0.49	***P *** **= 0.026**	*P *= 0.69	*P *= 0.48	*P *= 0.065	*P *= 0.37
Liver pFOXO1	Increased	Increased in males	*P *= 0.19	***P *** **= 0.002**	*P *= 0.24	*P *= 0.20	***P *** **= 0.003**	*P *= 0.072

### Glucose tolerance

**Figure 2:**
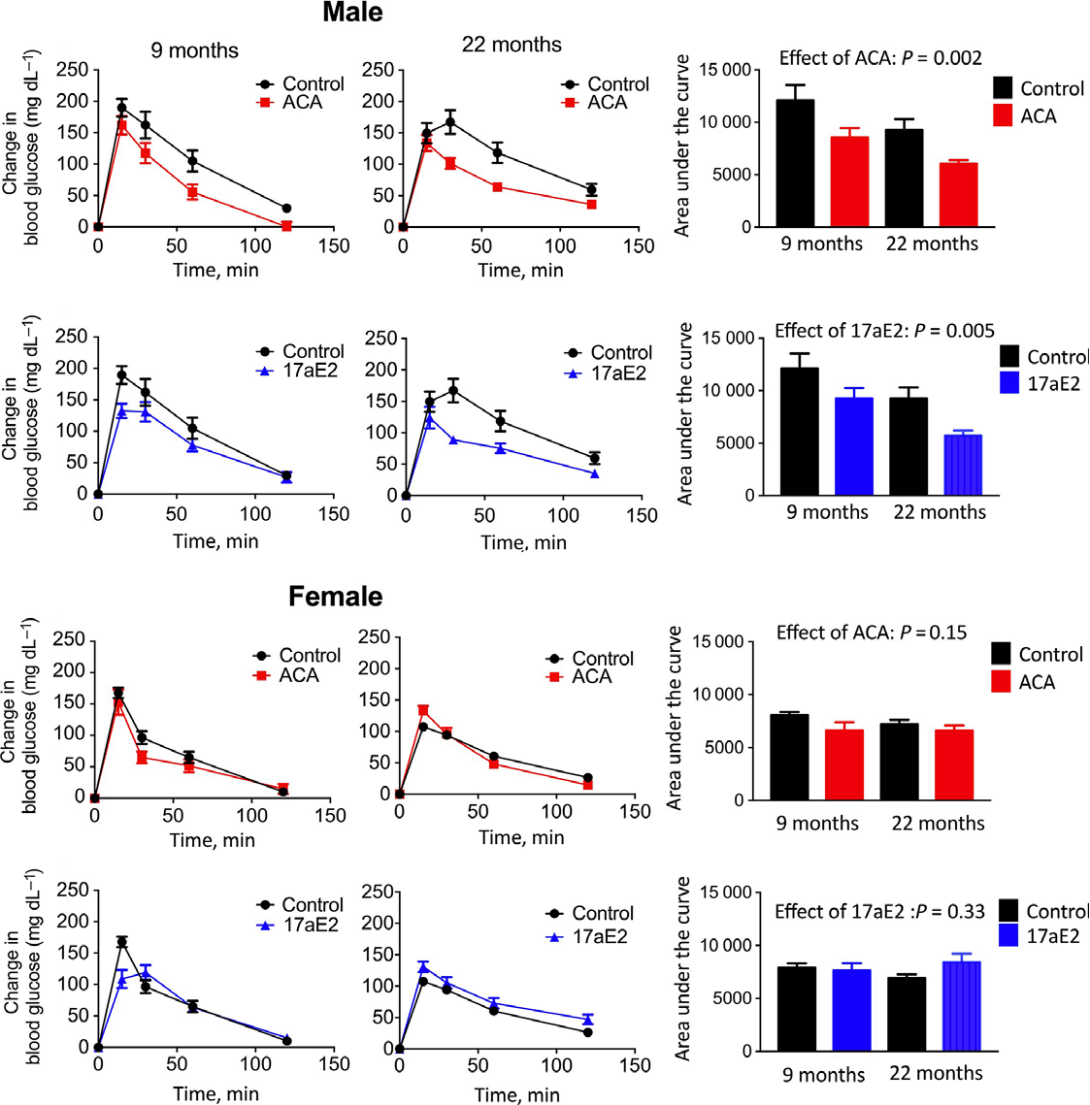
Enhanced glucose tolerance in males treated with ACA or 17aE2. Glucose tolerance tests were conducted in mice at either 9 or 22 months of age (*n *= 8–12 for each sex in each treatment group at each test point); see methods for details. Bar graphs on right show the area under the curve calculated by using glucose levels at T = 0 as a baseline, with *P*‐values presenting the drug effect in a two‐way ANOVA, including age as the second parameter, for each sex separately.

We conducted glucose tolerance tests to see whether these sex‐specific responses in insulin sensitivity lead to altered glucose tolerance with treatment. Male mice have in some studies been found to have decreased glucose tolerance compared to females (Stubbins *et al*., 2012; Varlamov *et al*., 2015), which may contribute to metabolic dysfunction in older males. As ACA leads to a consistent, sex‐independent elevation in glucose levels after fasting (Fig. 1E), likely to be due to the actions of ACA in slowing starch breakdown, we calculated glucose excursion following IP glucose, using glucose levels just prior to injection of a glucose bolus as a baseline when calculating area under the curve. Assessment of glucose excursion after either 5 or 18 months of treatment with ACA or 17aE2 revealed that both drugs increased the ability of males to remove administered glucose (and therefore reduce plasma levels following injection), while having no such effect on females (Fig. 2; Table 1). The effect of treatment on glucose excursion at the measured time points also differed according to sex at the 30‐, 60‐, and 120‐minute time points, for both ACA and 17aE2 (sex by treatment interaction: *P* < 0.05 at each time point for each drug). There is also a significant effect of age in this analysis, with older animals appearing to have improved glucose tolerance (*P* = 0.003 across the whole dataset), although we are cautious of interpretation of this result as the two age groups were tested approximately a year apart.

### Sex‐specific alterations in mTOR signaling after exposure to ACA or 17aE2

Alterations in mTOR signaling have been associated with sex differences in lifespan extension and can differentially influence glucose homeostasis in males and females. Reduced mTORC1 signaling extends lifespan to a greater degree in females (Lamming *et al*., 2012; Miller *et al*., 2014; Garratt *et al*., 2016), while genetic inhibition of mTORC2 reduces male lifespan without noticeably affecting females (Lamming *et al*., 2014b). At least some of these sex effects have been suggested to be attributable to the negative effects of reduced mTORC2 on glucose homeostasis in males, as impaired activation of this complex can reduce glucose tolerance to a greater degree in male mice (Lamming *et al*., 2012, 2014b). Alterations in the activity of both mTOR complexes have also been observed in other mouse models of lifespan extension: Snell dwarf and growth hormone receptor‐deficient mice show lowered mTORC1 signaling and increased mTORC2 signaling, in both sexes, which is consistent with the lifespan extension observed in both sexes in these models (Dominick *et al*., 2014).

**Figure 3:**
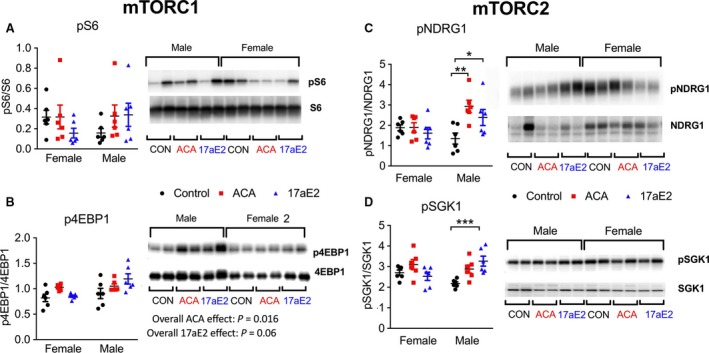
Activation of mTOR substrates in livers of mice treated with 17aE2. From liver samples taken at 12 months of age (*n *= 6 per group per sex). Statistics showing overall effects (B) represent the *P*‐value for a treatment parameter in a two‐way ANOVA that also included a parameter for sex. * represents *P* < 0.05; ** represents *P* < 0.01; *** represents *P* < 0.005 from a Student's *t*‐test conducted on the data separately from each sex.

Given the potential roles of mTOR signaling in sex differences in aging and metabolism, we examined the phosphorylation status of several mTOR substrates in livers of fasted males and females that had been treated with ACA and 17aE2 for 8 months (i.e., tested at 12 months of age). S6 and 4EBP1 are substrates downstream of mTORC1. S6 phosphorylation did not significantly change with either ACA or 17aE2 (Fig. 3A). In contrast, and surprisingly, phosphorylation of 4EBP1 was increased with ACA (*P* = 0.016 for ACA and *P* = 0.06 for 17aE2), and to a similar degree in males and females (Fig. 3B). We also note here that total 4EBP1 protein levels were reduced in females, but were unaffected by drug treatment in males (Table S1).

In contrast to the effects for mTORC1, the change in phosphorylation of mTORC2 substrate NDRG1 in response to either ACA or EST is sex specific (Fig. 3C; Table 1), with males showing an increase in NDRG1 phosphorylation in response to both drugs and females showing no change. A similar pattern is observed for pSGK1 in mice treated with 17aE2, also downstream of mTORC2, with males alone showing significantly increased activation in response to 17aE2 (Fig. 3D). For ACA, there is no significant interaction between sex and treatment (Table 1), with ACA increasing SGK1 phosphorylation in both males and females (effect across both sexes: *P* = 0.006). We note that this result for pSGK1 should be viewed cautiously, because the antibody used to detect pSGK1 at S422 is polyclonal, and in cell lysates has been reported to detect a rapamycin‐sensitive phosphorylated protein of a similar molecular weight (Garcia‐Martinez & Alessi, 2008). ACA and 17aE2 also led to decreased levels of total NDRG1 in liver in both sexes, and ACA also reduced levels of total SGK1 in a sex‐independent manner (Table S1).

### Alterations in substrate phosphorylation downstream of mTORC2

**Figure 4:**
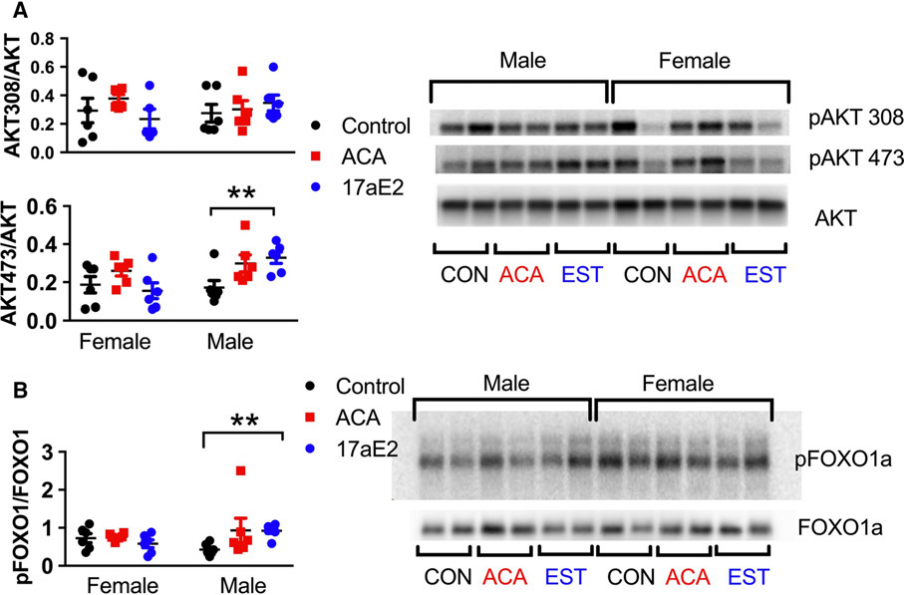
Sex‐specific regulation of AKT473 and FOXO1 phosphorylation with ACA and 17aE2. From liver samples taken at 12 months of age (*n *= 6 per group per sex). ** represents *P* < 0.01 from a Student's *t*‐test conducted on the data separately from each sex.

Akt is a major mTORC2 target involved in the regulatory responses to insulin. mTORC2 phosphorylates Akt at residue S473 but does not phosphorylate T308 (Kennedy & Lamming, 2016). Both ACA and 17aE2 increase Akt phosphorylation at S473 in males but do not affect T308 (Fig. 4A), consistent with elevated mTORC2 activity and enhanced insulin signaling. There is a significant sex by treatment interaction for mice treated with 17aE2 (Table 1), indicating that males and females show different changes in pAKT473 in response to 17aE2, with females showing no change with treatment. For ACA, the sex by treatment interaction is nonsignificant, but the main effect of treatment is significant (*P* = 0.015), indicating that both males and females show an increase in pAKT473 in response to ACA (Fig. 4A).

FOXO1 plays an important role in glucose metabolism. mTORC2 and the PI3K‐Akt/SGK1 pathway negatively regulate FOXO1 activity. mTORC2 activity leads to phosphorylation of FOXO1 at T24, which contributes to nuclear exclusion and inhibition of hepatic FOXO1 activity (Lamming *et al*., 2014a). We examined T24 phosphorylation of FOXO1 in whole tissue lysates, and observed that T24 phosphorylation is increased in males but not females treated with 17aE2 (Fig. 4B), with the significant interaction term indicating that males and females show a significantly different change in FOXO1 phosphorylation in response to treatment (Table 1). For ACA, there is no significant effect on FOXO1 phosphorylation, and there is no interaction between sex and treatment.

### Sex hormones underlying sex‐specific drug responses

Both testosterone and estrogens have been linked to sex differences in lifespan (Maklakov & Lummaa, 2013; Regan & Partridge, 2013; Austad & Bartke, 2015), and each of these hormones can influence glucose tolerance and insulin sensitivity (Geer & Shen, 2009; Legato, 2010). To test whether sex‐specific responses to ACA and 17aE2 were dependent on differences between males and females in adult life gonadal hormone production, we castrated males and ovariectomized females at 3 months of age, then treated them with ACA or 17aE2 from 4 months of age, that is, over the same time period as the sham‐operated animals presented above. Sham‐operated mice and those subjected to gonadectomy were produced, aged, and treated in parallel.

**Figure 5:**
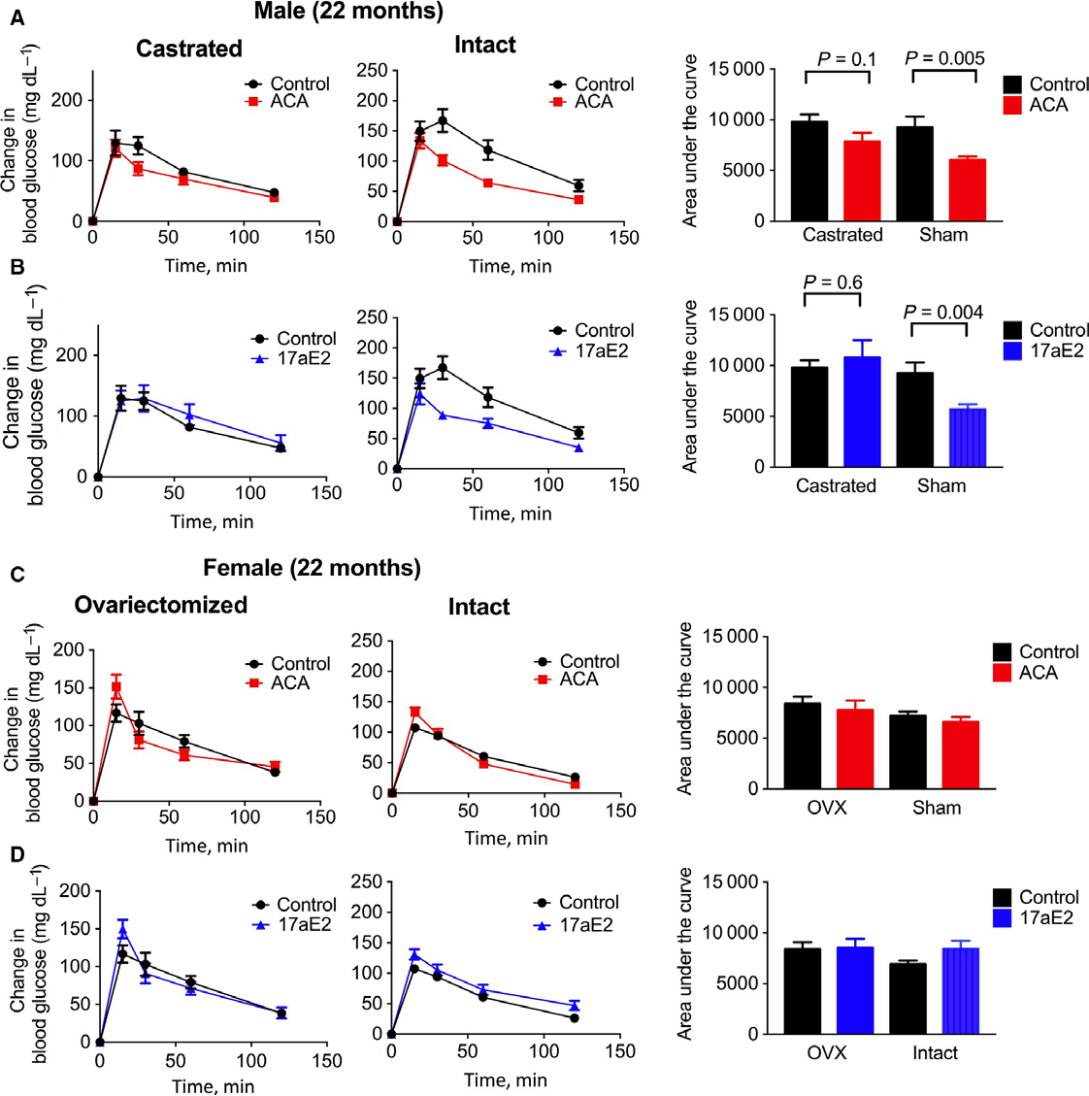
No improvement in glucose tolerance in castrated males treated with ACA or 17aE2. Glucose tolerance tests were conducted in mice at 22 months of age (*n *= 8–12 for each sex in each treatment group at each test point). Data from intact animals are replicated from Fig. 2 and included here for ease of comparison. See methods for details on GTT. Bar graphs on right show the area under the curve, with *P*‐values above bars representing the effects of drug treatment for either castrated or intact males, using a Student's *t*‐test.

In contrast to intact males, castrated males showed no significant improvement in their ability to clear glucose after ACA or 17aE2 treatment when tested at 22 months of age (Fig. 5). For glucose levels relative to baseline at each time point, we conducted two‐factor ANOVAs and tested whether there was an interaction between treatment (e.g., control or 17aE2/ACA) and surgical status (gonadectomized or intact) within each sex. A significant interaction term in such an analysis would demonstrate an effect of castration on the treatment response in male mice. The effect of 17aE2 on male glucose clearance is significantly altered by male castration, both at the 30‐ and at 60‐min time points (surgery*treatment interaction: 30 min: *P* = 0.019; 60 min: *P* = 0.019), showing that the male‐specific benefit in terms of improved glucose excursion with this drug is inhibited in castrated males. The effect of ACA on male glucose clearance is also significantly altered by male castration at the 60‐min time point (surgery*treatment interaction: *P* = 0.039), again showing that castration significantly diminishes male treatment responses. Sham‐operated females did not show an improvement in glucose tolerance with either drug treatment, and OVX mice were no different in this regard (Fig. 5).

### Reversal of mTORC2 signaling with castration and ovariectomy

**Figure 6:**
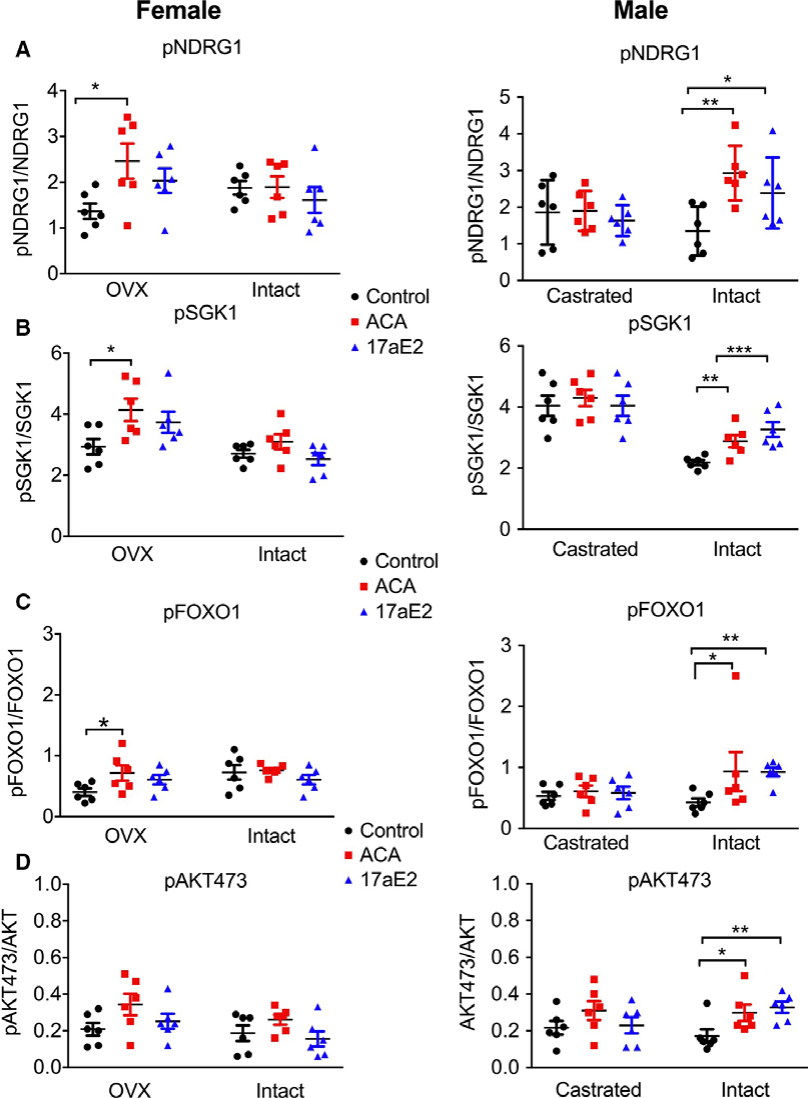
Sex‐specific regulation of mTORC2 substrates is mediated by gonadal hormones. Data from intact individuals are replicated from Figs 1 and 3, and shown here for ease of comparison. Results from samples collected from 12‐month‐old mice, *n *= 6 per group. *P*‐values were calculated using a Student's *t*‐test; see Table 1 for additional details. * represents *P* < 0.05; ** represents *P* < 0.01; *** represents *P* < 0.005.

To understand whether sex‐specific changes in hepatic mTORC2 signaling, AKT and Foxo1 phosphorylation with drug treatment were also reversed by gonadectomy, we evaluated phosphorylation of these substrates in castrated male and ovariectomized female mice that had been exposed to ACA or 17aE2. Sex‐specific activation of each of these substrates with 17aE2 is modulated by gonadectomy, and there is evidence that both male castration and female ovariectomy can influence treatment responses. Indeed, in a three‐way ANOVA, including sex (male or female), treatment (control or 17aE2), and surgery (gonads removed or sham surgery), for each substrate, there is a significant sex*treatment*surgery interaction highlighting the effect of gonadal hormones in modulating sex‐specific treatment responses in the liver (*P* = 0.05 for pAKT 473; *P* = 0.007 for pNDRG1; *P* = 0.001 for pFOXO1; *P* = 0.002 for pSGK1). The increase in phosphorylation of these substrates that was seen in intact males with 17aE2 is not observed in castrated males (Fig. 6), revealing that male gonadal hormones are required for male‐specific treatment responses, and there is a significant surgery by treatment interaction within males for pSGK1 and pFOXO1 (Table 1). There is also some evidence that female ovariectomy can modulate the female response to 17aE2, as there is a significant surgery by treatment interaction within females for pNDRG1 (Fig. 6).

For ACA, we observed sex‐specific phosphorylation only of NDRG1, with the other substrates responding similarly in both sexes. For this substrate, we also found a significant sex*treatment*surgery interaction (*P* = 0.002), with two‐way ANOVAs within each sex indicating that both male castration and female ovariectomy influence treatment responses (Table 1). Ovariectomized females show a significant increase in phosphorylation of NDRG1 with ACA treatment (Fig. 6A), similar to intact males, while castrated males show no change with treatment, thus similar to the lack of response seen in intact females.

## Discussion

Our results show that ACA and 17aE2, which lead to mouse lifespan extension principally in males, also produce male‐specific improvements in glucose tolerance and elevations in hepatic mTORC2 activity. Females, which do not show lifespan extension with 17aE2, and show only a 5% improvement in median lifespan with ACA, do not show improved glucose tolerance when treated with either drug, and show less activation of mTORC2 substrates with treatment, particularly for 17aE2, consistent with the lack of any survival effect for 17aE2 in female mice.

The data on sex‐specific changes in mTORC2 signaling and glucose tolerance are consistent with the recent observation that genetic inhibition of mTORC2, either globally or specifically in the liver, reduces lifespan specifically in males, without affecting females (Lamming *et al*., 2014b). Both increases and decreases in male mouse lifespan, therefore, seem to be linked to alterations in hepatic mTORC2 function, such that increased mTORC2 activity is associated with male lifespan extension, while inhibiting mTORC2 activity reduces male survival. Activation of mTORC2 is involved in the regulation of glucose uptake in response to insulin (Kennedy & Lamming, 2016). Elevated mTORC2 activity may promote hepatic responsiveness to insulin and could contribute to the enhanced glucose tolerance with drug treatment observed in this sex. If male lifespan is more sensitive to transient or postprandial perturbations in glucose homeostasis than that of females, this sex specificity could contribute to the differences in longevity effects. Alternatively, this apparent relationship between male lifespan and mTORC2 signaling could be related to some other function or regulator of mTORC2, including lipids, leptin, or altered activity of TSC2. Understanding the causal factors underlying this relationship, and the impact of mTOR signaling in control of sex‐specific metabolism and pathology in other tissues types under periods of both feeding and fasting, may provide a significant insight into the molecular signals controlling sexual dimorphism in aging.

The pathways through which 17aE2 improves male glucose tolerance remain to be defined. 17aE2 binds only weakly to classical estrogen receptors (Perez *et al*., 2005), although it can still elicit some uterotrophic effects in OVX females (Strong *et al*., 2016). Some of the metabolic effects of ERα activation also occur through protein–protein interactions that are independent of nuclear translocation of the E2–ER complex (Gupte *et al*., 2015). The activation of these responses requires a much lower binding affinity of estrogens to ERα (Madak‐Erdogan *et al*., 2016) and thus could conceivably occur in response to 17aE2. 17aE2 crosses the blood–brain barrier and can have neuroprotective effects in mouse models of ischemia (Perez *et al*., 2005), and 17aE2 can bind to a brain‐specific estrogen receptor ER‐X (Toran‐Allerand *et al*., 2002, 2005). It was recently shown that both ACA and 17aE2 reduce age‐dependent hypothalamic inflammation in mice and that these effects are much stronger in males (Sadagurski *et al*., 2014). Regulation of glucose homeostasis and tissue‐specific insulin signaling in drug‐treated mice might therefore involve CNS regulation of energy metabolism, as reduced hypothalamic inflammation can improve metabolic dysfunction (Cai & Liu, 2011) and even increase lifespan in mice (Zhang *et al*., 2013).

Our results further reveal that sexually dimorphic responses to these drugs are influenced by both male and female gonadal hormones, and typically in opposite directions. Castrated males do not show improvements in glucose tolerance with either ACA or 17aE2 and do not show increased activity of hepatic mTORC2. Thus, male gonads, probably via testosterone production, contribute to these sexually dimorphic metabolic responses, with castrated males showing the lack of drug response typical of intact females. Strikingly, OVX causes females to show some phenotypic responses to treatment that are observed in intact males, but not in intact females. Follow‐up studies in which testosterone or 17‐β estradiol are administered throughout adult life to intact or gonadectomized mice would be technically quite difficult. These would require repeated injections, which can themselves potentially lead to effects on health and hormone status, and would require duplication of age‐related changes in hormone levels, which would not be able to replicate circadian and environmental influences in hormone levels. It may be more feasible to explore these issues using mice with mutations in receptors for androgens and estrogens, either globally or in specific cell types. Nonetheless, these results suggest that both male and female gonadal hormones contribute to sex differences in metabolic function and intracellular signalling in response to ACA and 17aE2. It would be of considerable interest to evaluate lifespan effects of both drugs in castrated males and OVX females, and the development of other aspects of age‐associated metabolic dysfunction and pathology that are differentially affected by treatment in each sex (e.g., Harrison *et al*., 2014). Our work suggests that castrated males would show little or no lifespan benefit from either drug and that OVX might allow females to benefit from one or both of these interventions. Such data would be of particular use as a guide toward developing drugs, in these classes, that might slow aging or have other health benefits in both men and women.

The consistent effect of castration in inhibiting male responses to drug treatment could occur via various postulated processes. Testosterone, or a protein/phenotype expressed in response to testosterone, might alter bioactivity, conversion to bioactive forms, or cellular responsiveness to either drug. For example, many genes involved in xenobiotic metabolism show sexually dimorphic expression and are partially controlled by the continuous production of sex hormones in adult life (Waxman & Holloway, 2009). For ACA, however, the location of drug action is thought to be in the small intestine, where ACA inhibits alpha glucosidase, slowing the breakdown of complex carbohydrates to absorbable glucose. This primary effect of ACA appears to occur in a sex‐independent manner, because fasting glucose levels are elevated to a similar degree in both sexes. Thus, sex‐ and hormone‐dependent differences in ACA responses presumably reflect consequences of alterations in responses to transient postprandial glucose excursions, rather than to the direct effects of ACA itself on glucosidase function.

The lack of drug effects on castrated males, and the facilitation of drug effects by OVX in females, may reflect opposing effects of sex hormones on aspects of physiology linked to lifespan. Male castration extends male lifespan in various species (Brooks & Garratt, 2017), including situations in which castration is delayed until after puberty (Asdell *et al*., 1967; Drori & Folman, 1976), while OVX has been reported to reduce female mouse survival when conducted in adulthood (Benedusi *et al*., 2015). Our work shows that at least some of the sex‐specific effects of ACA and 17aE2 reflect actions of gonadal hormones in adult, that is, postpubertal mice, and do not reflect sexual dimorphisms established prior to 3 months of age. Adult castration and OVX have also been reported to have opposing effects on specific cell responses to insulin, at least in mouse adipocytes, which become more insulin sensitive in castrated male mice, while OVX has the opposite effect in females (Macotela *et al*., 2009). However, the observation that lifespan of males treated with 17aE2 exceeds that of both control‐ and 17aE2‐treated females (Strong *et al*., 2016), suggests that this treatment does not simply protect against some male dysfunction that reduces male lifespan in relation to that of normal females.

Our work does not establish whether the beneficial antiaging effects of ACA and 17aE2 require improved glucose handling and/or altered responses to insulin, in the liver, or in any other cell type. Comparison of glucose tolerance in mice tested at 9 or 22 months of age suggested that the older mice might have more effective glucose clearance, but this inference must be taken with great caution, because the two groups were tested approximately one year apart, making direct comparisons hazardous. Nonetheless, ACA and 17aE2 do not appear to specifically protect against age‐associated declines in glucose tolerance, as observed in some lifespan models in C57BL/6 mice (Blüher *et al*., 2002; Selman *et al*., 2008), where glucose levels in older animals remain consistently high following an administered glucose bolus. Rather, we find that ACA and 17aE2 produce male‐specific improvements in glucose tolerance consistent across most of adult life. How such changes in glucose tolerance and underlying insulin signaling might be linked to improved male survival requires further investigation. It is also notable that although ACA‐treated females appear to show a slight reduction in glucose tolerance and insulin sensitivity compared to untreated female controls, this sex still shows a significant, albeit smaller, lifespan extension in response to ACA. At least part of the lifespan‐extension effect in ACA‐treated females is therefore independent of improved glucose tolerance, although hepatic phosphorylation of SGK1 and AKT was increased in both sexes, which might promote insulin signaling specifically at this site. We observe that both plasma IGF1 and adiponectin concentrations are reduced with ACA treatment, in a sex‐independent manner, showing that additional/complementary endocrine pathways are modulated by ACA. The reduction in plasma adiponectin contrasts with effects observed in other mouse lifespan‐extension models, including GHR‐knockout (Berryman *et al*., 2004) and DR‐treated mice (Cawthorn *et al*., 2014), which show increased circulating adiponectin. In this study, we assessed total plasma adiponectin, but it has recently been shown that changes specifically in the high molecular weight isoform of adiponectin can occur with DR (Miller *et al*., 2017), and this isoform may provide specific metabolic benefits. Reduced circulating IGF1 is a potential candidate linking to ACA to increased female lifespan, as reduced IGF1 signaling can extend mouse lifespan, with preferential survival benefits in females (Garratt *et al*., 2017). Greater understanding of the physiological and underlying hormonal causes for sexual dimorphism in lifespan extension with ACA and 17aE2, and for that matter reduced mTOR signaling and IGF1 signaling (which preferentially extend female lifespan), could provide significant insights into sexual dimorphism in the aging process and provide guidance to the development of drugs that are confer health benefits in one or both sexes.

## Experimental procedures

UM‐HET3 mice were produced as previously described (e.g., Miller *et al*., 2014; Strong *et al*., 2016). The mothers of the test mice were CByB6F1/J, JAX stock #100009, whose female parents are BALB/cByJ and whose male parents are C57BL/6J. The fathers of the test mice were C3D2F1/J, JAX stock #100004, whose mothers are C3H/HeJ and whose fathers are DBA/2J. Mice in breeding cages received Purina 5008 mouse chow, and weaned animals were fed Purina 5LG6.

Mice were housed as previously described (e.g., Miller *et al*., 2014; Strong *et al*., 2016) in plastic cages with metal tops, using ¼‐inch corn‐cob bedding (Bed O'Cobs, produced by The Andersons, PO Box 114, Maumee, OH, USA). Mice were given free access to water, using water bottles rather than an automated watering system. Mice were housed in ventilated cages and were transferred to fresh cages every 14 days. Temperature was maintained within the range of 21–23°C.

### Surgical procedures

At 3 months of age, all animals went through castration, ovariectomy, or a sham procedure. All animals were anesthetized by injection of 250 mg kg^−1^ tribromoethanol, and given a single preoperative injection of the analgesia carprofen, at 5 mg kg^−1^.

### Castration and sham castration

After surgical preparation, an incision was made in the caudal end of each scrotal sac, the testicle was pulled through the incision by gentle traction, and the blood vessels, vas deferens, and deferential vessels were clamped and sutured. The incision was closed with tissue adhesive. For sham surgery, the testicles were exteriorized and then replaced in the scrotum, without being ligated or excised.

### Ovariectomy or sham ovariectomy

After surgical preparation, an incision was made on the left side perpendicular to the vertebral column approximately midway between the iliac crest and the last rib. The ovarian fat pad was grasped and exteriorized. The pedicle under the ovarian blood vessels and fat pad under the ovary were grasped and crushed, the pedicle cut on the ovary side and the ovary removed, and the blood vessels tied with absorbable suture. The abdominal wall was closed with absorbable suture and skin was closed with staples. The procedure was then repeated on the opposite side. For sham ovariectomy, animals underwent the same surgical procedure, but the ovary and fat pad were exteriorized and replaced without being excised.

### Diets

At 4 months of age, animals in different sibling groups were randomly allocated to control, ACA, or 17aE2 treatment. Animals in the control group remained on the 5LG6 diet, while animals allowed allocated to ACA or 17aE2 had their diet switched to one of these experimental diets.

All diets were prepared by TestDiet, Inc., a division of Purina Mills (Richmond, IN, USA). Purina 5LG6 food contained each of the test substances and was used as the control diet. 17aE2 was purchased from Steraloids Inc. (Newport, RI, USA) and mixed at a dose of 14.4 milligrams per kilogram diet (14.4 ppm). Acarbose was purchased from Spectrum Chemical Mfg. Corp., Gardena, CA, USA, and was mixed at a concentration of 1000 mg of ACA per kilogram of diet (1000 ppm). These methods followed those used by the NIA Interventions Testing Program.

### Metabolic analysis

Intraperitoneal glucose tolerance tests were performed on mice fasted for 16 h overnight. Blood glucose levels were measured using a Glucometer Elite (Bayer), after which mice were injected intraperitoneally (ip) with D‐glucose (2 g kg^−1^), and blood glucose levels were monitored over 120 min. For insulin tolerance tests, mice were fasted for a four‐hour period in the light cycle before ip injections of insulin (0.8 U kg^−1^; Humulin R) diluted in sterile saline. Blood glucose concentrations were measured at the indicated time points.

Blood insulin, leptin, and total adiponectin levels were determined in plasma collected after an 18 hour fast using ELISA kits from Crystal Chem (Downers Grove, IL, USA). Blood IGF1 levels were assessed in plasma using the Mouse/Rat ELISA kit from ALPCO (Boston, MA, USA).

### Hepatic mTOR signaling

Livers were harvested during the morning, from 12‐month‐old mice after 18 h of fasting. Tissues were frozen with liquid nitrogen and stored at −80°C. Tissues were processed, whole‐cell lysates were obtained, and equal amounts of protein were loaded for Western blot analysis. Antibodies and phospho‐specific rabbit antibodies were purchased from Cell Signaling (pAKT 308: 9275; pAKT 473: 4060; total AKT: 9272; total FOXO1: 2880; pFOXO1 T24: 9464; total S6: 2217; pS6: 2211; p4EBP1: 2855; total 4EBP1: 9644; total NDRG1: 9408; pNDRG1: 5482 – www.cellsignal.com), Santa Cruz (pSGK1: 16745 – www.scbt.com), and Genetex (SGK1: 61249 – http://www.genetex.com).

### Statistics

Statistics were carried out in SPSS version 22. Data from animals treated with ACA and 17aE2 were analyzed separately, but the same control animals were used in both sets of analysis. For each measured parameter, we conducted a two‐factor ANOVA, using the general linear model function and a full factorial model, which included an effect of treatment (comparing control to either ACA or 17aE2), an effect of sex (male or female), and an interaction between sex and treatment. When testing for the effect of gonadectomy on treatment responses within each sex, we included an effect of treatment, an effect of surgery (gonadectomized or not), and an interaction between surgery and treatment. For those parameters that suggested there could be an effect of both male castration and female ovariectomy (*P* < 0.1 for the two‐way interaction term) on sex‐specific treatment responses, we conducted three‐way ANOVAs across data from both males and females of both surgical status, including fixed effects of sex, treatment, and surgical status, and interaction terms between each. Data were transformed where necessary to conform to assumptions of normality.

## Funding

This work was supported by grants from the Glenn Foundation for Medical Research, plus the National Institutes for Health AG024824 and AG022303. Michael Garratt also acknowledges support from the Michigan Society of Fellows.

## Conflict of interest

None declared.

## Supporting information


**Table S1** Parameter effects for changes in total protein levels of mTOR substrates in mice treated with ACA or 17aE2.
