# Supplementary material for: Sex differences in lifespan extension with acarbose and 17‐α estradiol: gonadal hormones underlie male‐specific improvements in glucose tolerance and mTORC2 signaling
Source: Aging Cell. 2017 Aug 22;16(6):1256–66. doi: 10.1111/acel.12656 (PMC5676051; doi:10.1111/acel.12656)
Supplement: Supplementary file 1 — Table S1 Parameter effects for changes in total protein levels of mTOR substrates in mice treated with ACA or 17aE2. [file ACEL-16-1256-s001.docx]

Table S1 Parameter effects for changes in total protein levels of mTOR substrates in mice treated with ACA or 17aE2.

| Parameters | 4ebp1/beta tubulin | | S6/beta tubulin | | NDRG1/beta tubulin | | SGK1/beta tubulin | |
| --- | --- | --- | --- | --- | --- | --- | --- | --- |
|  | P value | Direction | P value | Direction | P value | Direction | P value | Direction |
| Acarbose |  |  |  |  |  |  |  |  |
| Sex | P = 0.14 |  | P = 0.20 |  | P = 0.009 |  | P = 0.27 |  |
| Drug | P = 0.003 |  | P = 0.15 |  | P = 0.011 |  | P = 0.01 |  |
| Sex*Drug | P = 0.065 |  | P = 0.33 |  | P = 0.37 |  | P = 0.22 |  |
| Effect in males | P = 0.81 |  |  |  |  | Decreased |  | Decreased |
| Effect in females | 0.014 | Decreased |  |  |  | Decreased |  | Decreased |
|  |  |  |  |  |  |  |  |  |
| EST |  |  |  |  |  |  |  |  |
| Sex | P = 0.27 |  | P = 0.38 |  | P = 0.19 |  | P = 0.12 |  |
| Drug | P = 0.88 |  | P = 0.47 |  | P = 0.009 |  | P = 0.11 |  |
| Sex*Drug | P = 0.81 |  | P = 0.20 |  | P = 0.57 |  | P = 0.092 |  |
| Effect in males |  |  |  |  |  | Decreased |  |  |
| Effect in females |  |  |  |  |  | Decreased |  |  |
